# Biological noise to get a sense of direction: an analogy between chemotaxis and stress response

**DOI:** 10.3389/fgene.2014.00052

**Published:** 2014-03-13

**Authors:** Vera Pancaldi

**Affiliations:** Structural Computational Biology, Spanish National Cancer Research Centre (CNIO)Madrid, Spain

**Keywords:** biological noise, heterogeneity, chemotaxis, stress response, adaptation

## Introduction

From the earliest studies on bacteria (Spudich and Koshland, 1976; Elowitz et al., 2002), it was soon understood that biological processes are often dominated by the stochasticity that pervades the physical world. Biological noise, here defined as the substantial cell-to-cell variation that is observed in populations of genetically identical cells, is more and more recognized as an important factor in biology, thanks to the improvement of single cell analysis techniques. The stochasticity of physical phenomena that underlie cellular processes plays an important role in the origin of noise. Noise manifests itself at different levels. At the smallest scales, the random motion of molecules affects basic cellular processes such as transcription, translation, and signal transduction. At a higher level, the topology and connectivity of biological regulatory circuits is often such that stochastic behavior is observed in the levels of specific factors. Specifically, the topology of regulatory networks can feature multiple types of feedback and feed-forward loops, which create multistability in the system and can amplify the fluctuations originating from molecular noise (Burda et al., 2011). Both of these sources of noise can result in the existence of multiple stable states in cells, each defined by specific gene and protein levels. In isogenic cellular population this produces heterogeneity amongst cells and in time as stochasticity forces transitions between the different stable states (Levine et al., 2013; Sanchez and Golding, 2013). Although there is a clear difference between noise, which does not have a unique definition, and heterogeneity at the population level, the two phenomena are closely connected (Huang, 2009).

At first sight, the complexity of biological behavior would seem to require the strictest control of underlying processes, suggesting a constant evolutionary fight against the inherent disadvantages of noise. This is certainly the case, for example in the evolution of accurate proof-reading mechanisms that ensure faithful DNA replication. However, recent results also support the view that controlled levels of stochasticity are important in cells, proposing noise as a selectable trait. Already in 2004 Raser and O'Shea identified mutations both acting in cis and trans that affected the levels of gene expression noise, which is viewed as an evolvable trait (Raser and O'Shea, 2004). Ansel and colleagues later showed that the levels of expression noise in a single gene can be a heritable trait and they identified three Quantitative Trait Loci associated to it (Ansel et al., 2008). The adaptive importance of noise is exemplified in the concept of bet-hedging, according to which cells maximize their chance of survival by exploring different states in a random fashion (Fehrmann et al., 2013; Viney and Reece, 2013; Yvert et al., 2013).

The positive aspects of noise manifest themselves at many levels at which noise acts. At the molecular level, a control strategy based on dynamic equilibrium of stochastic events, similar to a thermostat regulating temperature, offers more robustness and tunability in processes such as mRNA production and translation (Shalem et al., 2008; Salari et al., 2012; Sanchez and Golding, 2013).

At the level of regulatory networks, topologies, and logic relations allowing oscillations and multistability contribute to higher adaptability of the organism to changing environments. It was recently shown that stress in yeast provokes a clear change in network topology, which can promote phenotype differentiation within a population (Mihalik and Csermely, 2011; Lehtinen et al., 2013). We can hypothesize that the observed loosening of the network could serve to diversify phenotypes in the different cells.

Single cell monitoring of phenotypes in tissues suggests a high degree of heterogeneity even in multi-cellular organisms (Paszek et al., 2010). An important factor in cell-cell communication, NFkB (nuclear factor kappa-light-chain-enhancer of activated B cells), undergoes continuous cycles of nuclear localization, such that in a population only a fraction of the cells at any time will have high levels of this transcription factor's activity (Ashall et al., 2009). In the overall context of a tissue, this heterogeneity in activation levels of NFkB contributes to the maintenance of homeostasis and to tissue responsiveness. Thus, even at the tissue level, the presence of fluctuations in time and across cellular populations serves as a detection tool that ensures fast reaction to any changes in the tissue's condition (Paszek et al., 2010; Levine et al., 2013).

Although the connections between noise at these different levels are still to be elucidated, noise pervades biological life forms across evolutionary time and there does not seem to have been a strong negative evolutionary pressure to eliminate it, with a few exceptions (Lehner, 2008).

A possible generalized interpretation of the above observations is that fluctuations, and hence noise at multiple levels, are instrumental in granting a robust control strategy to organisms enabling them to adapt to changing environments. We will try to substantiate this claim through two examples that lead us to make an analogy between bacteria aiming to reach a source of food and a population of yeast aiming to reach a state of optimal growth in a varying environment.

## Chemotaxis: charting the map with biased random walks

Bacteria can identify sources of food and repellent substances based on the presence of just a few molecules in a background concentration spanning many orders of magnitude (Wadhams and Armitage, 2004). It was discovered that bacteria can either swim in a specific direction or tumble, that is rotate on themselves, and hence randomly change their direction. These two different actions are determined by the sense of rotation of their flagella, which is the output of a complex signaling network. Combining the two types of motion, evolution has selected a strategy that allows bacteria to explore their environment in search for food, performing what is effectively a random walk biased by the concentration field. When the bacterium swims across increasing food concentration, the tumble motion is suppressed, whereas, when there is no clear concentration gradient, tumbling is favored and a new random direction is chosen. A closer look at the mechanism behind this phenomenon reveals that random motion, combined with signal processing and feedback can explain this behavior (Sourjik and Wingreen, 2012).

Thus, stochastic behavior is at the origin of cells' ability to navigate their environment. We can say that the cell is building a model of the “world” outside and with time it keeps re-adjusting it. Each stochastic tumble-swim event brings an occasion to confront the model with the environment, through detection of the actual chemical concentration gradient. Moreover, when an entire population of bacteria is present, stochastic mechanisms ensure that each individual will have a sufficiently different behavior from the others to efficiently explore the entire space (Korobkova et al., 2004; Emonet and Cluzel, 2008). This strategy is likely to be under selection, as the capacity to navigate environments, find food and avoid repellents is a clearly important trait for bacteria and is even conserved in the immune system (Luster, 2001).

## Mechanisms of stress response and adaptation in yeast

Yeast cells perform very broad rearrangements of their transcriptional program upon stress treatment (Gasch et al., 2000; Gasch, 2007; Berry and Gasch, 2008; Chen et al., 2008; Lackner et al., 2012) that allow the cell to produce the correct amount of proteins that it needs to deal with challenges, including de-toxifying agents and chaperones, and regulate its cell cycle. Growth and cell division genes are down-regulated concomitantly with the temporary arrest of the cell cycle, while genes involved in stress protection are up-regulated (Pancaldi et al., 2010). It is generally observed that each cell can either choose to grow faster, reducing its resilience to changes in the conditions, or grow slower, allowing it to better survive external changes (López-Maury et al., 2008).

In recent experiments with budding yeast, stress was seen to increment morphological variability at the population level, possibly through an effect related to HSP90 (Hsieh et al., 2013), an important chaperone buffering the effects of multiple genetic mutations (Rutherford and Lindquist, 1998). Investigations on single cell growth rates in budding yeast propose that keeping a wide distribution of values for this parameter can be exploited as a stress defense mechanism against severe heat stress (Levy et al., 2012). Sequence variants in yeast are known to affect gene expression variability (Fehrmann et al., 2013) but, importantly, in this case, the presence of a distribution of growth rates in the population is found not to depend on the genetic make-up of the single cells, as the character is not heritable. Instead, it is attributed to a large number of epigenetic states which determine the growth rate and hence affect the propensity to resist stress in each cell. This was verified by following the progeny of cells that displayed a particular low or high growth rate and observing the cells revert to the original distribution after a few tens of generations (Levy et al., 2012). Particular types of stress, like nitrogen starvation, even prompt yeast to perform meiosis and produce spores, more resistant cells that can survive in a dormant state until external conditions have improved (Su et al., 1996; Otsubo and Yamamoto, 2012). Meiosis involves homologous recombination, which could be interpreted as a further attempt to maximize the variability within the population. Once again the presence of a wide distribution of values of a specific character, in this case of growth rate, confers an advantage to the whole population, which will thus avoid extinction when its perfectly adapted fast growing cells are killed.

## Stress response variability as mapping in time

**Figure 1:**
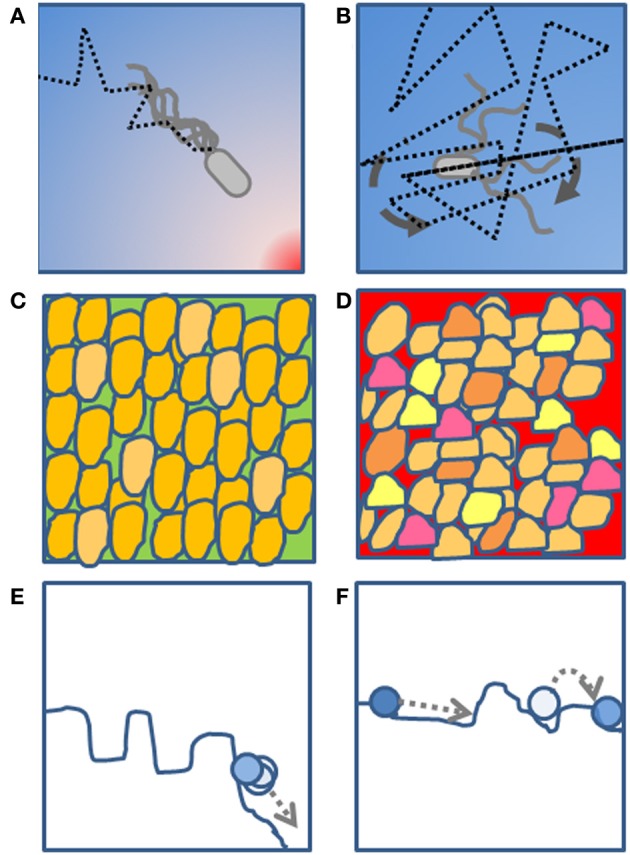
**A schematic diagram of the proposed analogy. (A)** A bacterium moving toward a source of food through a random walk biased by the chemical concentration gradient. **(B)** A bacterium tumbling most of the time and performing a non-biased random walk in the absence of a chemical gradient. **(C)** A phenotypically homogeneous yeast population growing in a rich medium in the absence of a perturbation. **(D)** A phenotypically heterogeneous yeast population growing under a chemical perturbation, where presumably the heterogeneity is a strategy to ensure survival of at least a few cells during the perturbation. **(E)** A fitness landscape with clear minima (valleys) and where the homogeneous population is coordinately descending toward the global minimum. **(F)** A flatter fitness landscape where, in absence of a clear gradient, the population explores various regions through heterogeneity in the single-cell phenotype.

Thus, stress response in cells is similar to chemotaxis, in the sense that predicting in which direction to go to get to the food corresponds to a yeast population deciding on whether growth conditions are going to get better or worse. In this case we can imagine the gradient of attractant or repellent to correspond to the increasing concentration of a chemical stress agent, for example hydrogen peroxide in a yeast culture. Until the cells are kept in good growth conditions, they simply optimize their growth rate, focussing their gene expression program and protein composition onto this task. In our analogy, this corresponds to a chemotacting cell, swimming almost straight toward a source of attractant. On the other hand, upon stress, the clear goal of optimal growth is substituted by the need to ensure survival. In the case of persistent insults, a variety of genes can be regulated to promote a more resilient state through differentiation of the single cell responses while changes in the interaction network topology further enhance it. In our analogy, this is the equivalent of the increased random tumbling motion in bacteria (Figure 1).

Just like bacteria in the absence of a clear gradient will freely spread in space maximizing their chance that at least one of them finds a gradient to follow, yeast populations in adverse environmental conditions will maximize cell-cell variability to more efficiently explore the fitness landscape.

These phenomena could be a consequence of the relaxation of control mechanisms that usually act to minimize the effects of noise, or there might be an evolved strategy to promote variability beyond that naturally present. In the first case, the resources the cell needs to keep the fluctuations at bay are not available in hostile environments or, in the second case, resources cost in guaranteeing variability at the population level has been evolutionarily selected as it is ultimately beneficial.

## Conclusion

In a sense, the diversification of gene expression, genetic and non-genetic features in single cells during stress corresponds to an exploration of the environment, which allows cells to achieve states that are better adapted to the external conditions. The few cells that will randomly achieve a phenotype that is appropriate for the environment will survive, at the expense of the ones whose phenotype is not favorable. Thus, it seems that the inevitable stochasticity of biological processes, accompanied by larger scale variability, could confer robustness to the entire colony in the face of external challenges. The whole population is navigating in a changing fitness landscape, similarly to a cell navigating a chemotactic gradient.

Two very different phenomena, chemotaxis and stress response, share the common feature of enabling the cells to orient themselves, in respectively spatially and temporally varying environments. Both mechanisms rely on the presence of two stochastic processes, the tumbling motion in chemotaxis and the single cell variability of gene expression in stress response. We thus support a very general interpretation of the pervasive noisy nature of biological processes, not only as a consequence of the underlying stochastic physical processes, but also as an important tool for cells that need to adapt to changing conditions.
